# Effects of Gestational Maternal Undernutrition on Growth, Carcass Composition and Meat Quality of Rabbit Offspring

**DOI:** 10.1371/journal.pone.0118259

**Published:** 2015-02-11

**Authors:** George K. Symeon, Michael Goliomytis, Iosif Bizelis, George Papadomichelakis, Olga Pagonopoulou, Zafeiris Abas, Stelios G. Deligeorgis, Stella E. Chadio

**Affiliations:** 1 Department of Physiology, Medical School, Democritus University of Thrace, Alexandroupoli, Greece; 2 Division of Animal Production, Department of Agricultural Development, Democritus University of Thrace, Nea Orestiada, Greece; 3 Department of Animal Breeding & Husbandry, Faculty of Animal Science and Aquaculture, Agricultural University of Athens, Athens, Greece; 4 Department of Anatomy & Physiology of Farm Animals, Faculty of Animal Science and Aquaculture, Agricultural University of Athens, Athens, Greece; 5 Department of Nutritional Physiology and Feeding, Faculty of Animal Science and Aquaculture, Agricultural University of Athens, Athens, Greece; University of Florida, UNITED STATES

## Abstract

An experiment was conducted in order to evaluate the effects of gestational undernutrition of rabbit does on growth, carcass composition and meat quality of the offsprings. Thirty primiparous non lactating rabbit does were artificially inseminated and randomly divided in three treatment groups: Control (C; fed to 100% of maintenance requirements throughout gestation, n = 10), early undernourished (EU; fed to 50% of maintenance requirements during days 7–19 of gestation, n = 10) and late undernourished (LU; fed to 50% of maintenance requirements during days 20-27 of gestation, n = 10). During the 4^th^ week of the gestation period, LU does significantly lost weight compared to C and EU groups (P<0.05). At kindling, C does produced litters with higher proportions of stillborn kits (P<0.05) while the total litter size (alive and stillborn kits) was not different among groups (10.7, 12.8 and 12.7 kits in C, EU and LU groups, respectively). Kit birth weight tended to be lower in the LU group. During fattening, body weight and feed intake were not different among offsprings of the three experimental groups. Moreover, the maternal undernutrition did not have any impact on carcass composition of the offsprings in terms of carcass parts and internal organs weights as well as meat quality of *L. lumborum* muscle (pH24, colour, water holding capacity and shear values) at slaughter (70 days of age). Therefore, it can be concluded that the gestational undernutrition of the mother does not have detrimental effects on the productive and quality traits of the offsprings.

## Introduction

There has been much research lately on the topic of developmental programming. This concept implies that a stimulus or insult acting during critical periods of fetus growth and development may result in developmental adaptations that permanently change the structure, physiology and metabolism of the offspring [1]. Among various stressors that have the ability to induce fetal programming, variation in the nutrient supply during fetal life and especially maternal undernutrition has been highlighted as a dominant cause.

Apart from effects in terms of susceptibility to cardiovascular or metabolic disease and to reproductive functioning which have been extensively studied [2, 3, 4], maternal undernutrition may also impact upon growth efficiency and body composition [5, 6]. It is known that in mammals the fetal period is crucial for both skeletal muscle [6] and adipose tissue development [7].

Modern rabbit breeding is based on a circular production system and the does are being inseminated every 35 or 42 days. In such a system, the females are always gestating, suckling or both gestating and suckling at the same time. These two physiological functions, especially lactation, are very costly in terms of energy [8]. Actually, a lactating doe suffers from a negative energy balance and considerable mobilization of body fat, because it’s feed intake cannot cover the requirements for both maintenance and milk production, even though it increases rapidly after kindling [9]. This energy deficit increases when females are concurrently pregnant and lactating [10]. In fact, lactation could have a detrimental effect on fetal growth [11]. These adverse effects of lactation on fetal growth can be partially simulated by the restricted feeding of the does during gestation [12].

Gestation in the rabbit may be regarded as having 3 stages: period between fertilization of the ovum or ova and implantation, the period of organogenesis, and the period of fetal growth. Implantation takes place 6–7 days after fertilization in the rabbit. The period of organogenesis is between days 7 and 18 or 19 (roughly the second third of the 30-day gestation period) and fetal growth is very rapid during the last third of gestation [13].

The effects of maternal undernutrition in rabbit offsprings have already been studied in terms of maternal weights and abortions [14, 15], litter size as well as offspring weight and survival rate [16, 17]. To our knowledge, no published study on the effects of maternal undernutrition on the offsprings’ production parameters can be found in rabbits. Therefore, the present study aimed to evaluate the effects of a 50% undernutrition of rabbit does imposed during two periods of gestation (early-stage of organogenesis and late-stage of rapid growth of fetuses) on growth as well as carcass and meat quality of the offsprings.

## Material and Methods

### Animal management and experimental design

Thirty Hyla Nouvelle generation primiparous non lactating hybrid does (*Oryctolagus cuniculus*) of similar age (7 months old) were purchased from a local breeding farm right after the weaning of their first litter. Upon arrival at the experimental facilities, the does were randomly allocated to three groups: Control (C, n = 10), early undernutrition (EU, at days 7–19 of gestation, stage of organogenesis, n = 10) and late undernutrition (LU, at days 20–27 of gestation, stage of rapid growth of fetuses, n = 10). The does were kept indoors in individual cages; the temperature ranged from +15 to +25°C and the light schedule was of 16 L: 8D. Prior to insemination, does were allowed to adapt to the new rearing environment for seven days. Specifically, initially they were fed *ad libitum* with the same pelleted diet that they received at the breeding farm which gradually was replaced with the experimental diet (Table 1). At day 0, they were artificially inseminated (sperm from Hylamax bucks) and they were induced to ovulate by injection of 10 μg of synthetic GnRH (Buserelin, Receptal, MSD Animal Health, UK).

**Table 1:** Ingredients and calculated chemical composition of the commercial diets used in the study.

	Type of diet		
Ingredient (% fed)	Gestation-lactation	Weaning	Fattening
Barley	25	11	17
Wheat middlings	14	20	23
Soybean meal 44%	9	5	7
Sunflower meal 28%	10	11	11
Alfalfa meal 18%	20	31	20
Sugar beet pulp	15	15	15
Soybean oil	1	1	1
Molasses	3	3	3
Calcium carbonate	1	1	1
Monocalcium phosphate	1	1	1
Salt	0.3	0.3	0.4
Lysine	0.1	0.1	0.1
Methionine	0.1	0.1	0.1
Threonine	0.05	0.05	0.05
Holine	0.15	0.15	0.15
Coccidiostat^1^	0.1	0.1	-
Vitamin and mineral premix^2^	0.2	0.2	0.2
Composition (% fed)			
Dry matter	91.26	90.96	90.77
Ash	7.82	9.08	8.52
Digestible Energy (MJ/Kg)	11.30	10.46	10.67
Crude Protein	16.58	16.38	15.67
Fat	4.27	4.13	4.20
Crude fiber	18.00	17.30	18.36

^1^ Robenidine 66 mg per kg of diet

^2^ The vitamin and mineral premix supplied per kilogram of diet: Vitamin A, 12.500 I.U.; Vitamin D_3_, 2.000 I.U.; Vitamin E, 75 mg; Vitamin K_3_, 3 mg; Vitamin B_1_, 2 mg; Vitamin B_2_, 6 mg; Vitamin B_6_, 3 mg; Vitamin B_12_, 20 mcg; Nicotinic acid, 60 mg; Pantothenic acid, 15 mg; Folic acid, 1,50 mg; Biotin, 250 mcg; Zn, 90 mg; Mn, 80 mg; Fe, 80 mg; Cu, 15 mg; I, 1,50 mg; Co, 1 mg; Se, 0,10 mg.

For the two following days after insemination, does of all groups were *ad libitum* fed a commercial gestation-lactation pelleted diet (Viozokat SA, Greece). From day 3 to day 6 of gestation all groups were fed to 100% of maintenance energy requirements while from day 28 to parturition they were fed *ad libitum*. From day 7 to day 28, control group was fed to 100% of maintenance energy requirements, whereas groups EU and LU were fed to 50% of maintenance energy requirements from day 7 to day 19, and from day 20 to day 27, respectively. The daily feed allowance (to 50 or 100% of maintenance energy requirements) was calculated individually for each doe once at the beginning of the experiment according to: a) the energy requirements of 430 KJ/day/kg bodyweight^0,75^ [18], b) the digestible energy content of the provided feed and c) the initial body weight of the does. The does had constant free access to water. Feed ingredients and chemical analysis of the diets are presented in table 1.

Parturition took place at 30.7 ± 0.3 days of gestation. At the second day *post partum*, kits were cross-fostered within nutritional groups in order to ensure equal litter sizes of 10 kits per doe. For the first 15 days of lactation, milk production of the does was measured by weighing the doe immediately before and after suckling. For this particular period, does were fed to 100% of maintenance energy requirements plus the milk production requirements which were calculated from the average daily milk production of the group [18]. For the rest of the lactation period, feed was provided for *ad libitum* intake. At weaning (35^th^ day of age), 32 rabbits per group (16 per sex) were randomly selected and kept indoors in individual cages with wire mesh floors, under the following environmental conditions (temperature: 26 ± 3°C; relative humidity: 60 ± 10%; lighting: 12 h/12 h light/dark cycle). Each cage was equipped with a metal feeder and a nipple drinker. Feed (commercial weaning diet, Viozokat SA, Greece; table 1) was provided *ad libitum* and rabbits had free access to water. At day 50 *post partum* the rabbits were allocated to a fattening feed (Viozokat SA, Greece; table 1). At day 70 *post partum* 16 rabbits per group (8 per sex) were randomly selected and slaughtered for the assessment of carcass and meat quality parameters.

### Measurements

**Body weights, feed intake and litter parameters**. At days 0, 7, 14, 21, 28 *pre partum* the does were individually weighted. Feed intake was recorded individually on daily basis. At parturition, litter weight and size (alive and stillborn kits) as well as the individual weight of the kits were recorded. Kits were then weighted weekly until slaughter. After weaning, the feed intake of the offsprings was recorded weekly.

**Carcass and meat quality**. At slaughter, live, cold carcass and reference weights (weight of the commercial carcass minus the head as well as the liver, kidneys and organs of chest and neck) as well as dressing percentage and internal organs weights (liver, perirenal fat, kidneys, and thoracic organs) were recorded. Twenty-four hours after slaughter carcass and meat quality was assessed. Specifically, the carcasses were cut into parts (front, mid, rear, fore and hind leg) and each part was weighted separately [19]. Furthermore, the fore and hint leg were dissected into meat, fat and bone and their weights were recorded. All meat quality measurements were made on the *Longissimus lumborum* muscle (LL). The pH 24 h postmortem (pH_24_) was measured at the level of the fourth lumbar vertebra of left side using a Sentron 1001 pH system model (Roden, the Netherlands), with the electrode inserted into the muscle. Meat colour was measured at the sixth lumbar vertebra section of the LL muscle on the internal surface of the muscle using a Miniscan XE chromameter (Hunterlab, Reston, VA) set on the L*, a*, b* system (L* = lightness, a* = redness, b* = yellowness). Percentage of released water (PRW) was studied in a sample of meat of the seventh lumbar vertebra. A sample of intact meat weighing 300 ± 5 mg was placed between two disks of Whatman No. 1 filter paper. The papers with the meat were placed between two Plexiglas plates and a load of 2.25 kg was applied for 5 min. The percentage of released water was calculated as the ratio of the percentage of weight of released water to intact meat [20].

For cooking loss and shear values measurements, the right loin of each carcass was weighed, placed in plastic bag, cooked in a water bath at 80°C for 1 hour, left under tap water for 15 min, and then left to cool in room temperature [21]. Cooking loss was estimated as the percentage of the weight of the cooked samples with respect to the raw ones. Warner-Bratzler (WB) measurements were carried out in samples that were obtained by cutting two parallelepipeds of 1×1 cm of cross section (1 cm^2^), and 2 cm-length along muscle fibre axis [22]. They were completely cut using a WB shear blade with a triangular slot cutting edge with the blade travelling at 100 mm/min to the sample [21]. Peak force values (N) were recorded. Intramuscular fat (IMF) was measured using a chloroform (Carlo Erba Reactifs–SDS, Val De Reuil, France): methanol (Merck, Darmstadt, Germany) 2:1 (v:v) solution and a cold extraction procedure [23].

**Statistical analyses**. Body weight and feed intake of the does, gestation length, litter size, litter weight and kit birth weight were analysed using a mixed model with dietary treatment as a fixed effect. For litter weight analysis, litter size was also included in the model as a covariate. The percentage of stillborn kits has been analysed using the Genmod procedure with binomial distribution and identity link function. The body weight and feed intake of the offsprings were analyzed using a mixed model for repeated measures with treatment, time, sex and their interactions as fixed factors as well as the doe as a random factor. Carcass weights and meat quality measurements were analyzed using a mixed model with treatment, sex and their interaction as fixed factors and the doe as a random factor. The effect of the offsprings’ sex was not significant and was therefore excluded from the models. Multiple comparisons were performed using Bonferroni’s multiple range test and significance was set at 0.05. Probability levels of 0.1>P>0.05 were considered as tendencies to differences. All results are presented as least square (LS) means and standard error of the means (SEM). Data were analyzed using the SAS/STAT statistical package [24].

### Ethic statement

This study was carried out in strict accordance with the guidelines of “Council Directive 86/609/EEC regarding the protection of animals used for experimental and other scientific purposes”. The protocol was approved by the Bioethical Committee of the Agricultural University of Athens (Permit Number: 21/06062014). All efforts were made to minimize suffering.

## Results and Discussion

### Body weight change and feed intake of the does

The weekly body weight change of the does in contrast to their initial body weight is presented in Table 2. There was no significant difference between the three experimental groups on the initial body weight. A significant loss of body weight was recorded for all the groups during the first week of gestation while in the second week, the EU group gained significantly less weight than both C and LU groups (P<0.05). Moreover, during the last week of gestation, the LU group lost around 115 g of body weight while C and EU groups gained weight (P<0.05).

**Table 2:** Effect of gestational undernutrition on body weight change (g), feed intake (g/doe/day) and kindling performance of does (n = 10).

		Treatment^1^			
	C	EU	LU	pooled SE	p-value
Initial Body weight (g)	4168	4677	4339	135	0.621
Body weight change (g)					
GD^2^ 7	-211	-288	-208	34	0.452
GD 14	185^a^	11^b^	193^a^	45	0.049
GD 21	41	-44	-10	35	0.786
GD 28	67^a^	176^a^	-115^b^	32	0.003
Feed intake (g/doe/day)					
GD 0-GD 6	126	122	132	6	0.903
GD 7-GD 19	115^a^	64^b^	119^a^	3	0.004
GD 20-GD 27	116^a^	126^a^	60^b^	3	0.029
GD 28-GD 31	300	337	288	10	0.518
Kindling performance					
Gestation length (d)	30.7	30.8	30.7	0.3	0.946
Abortions	-	2	-	-	-
Litter size	10.7	12.8	12.7	1.5	0.346
Litter weight (g)	545	584	516	32	0.443
Ratio of stillborn (%)	17.8^b^	0.0^a^	2.6^a^	3.2	0.002
Kit Birth weight (g)	57.1	55.7	48.1	3.3	0.064

^a,b^ Values within a row with different superscripts differ significantly at *P*<0.05.

^1^ C = does fed to 100% of maintenance energy requirements (n = 10); EU = does fed to 50% of maintenance energy requirements from day 7 to day 19 of gestation (n = 8); LU = does fed to 50% of maintenance energy requirements from day 20 to day 27 of gestation (n = 10).

^2^ GD = Gestation day.

The relevant weight loss that was recorded during the first week of gestation in all the experimental groups must be related to both, adaptation stress from moving from the breeding farm to the experimental station, and the reduction in feed intake when transitioning from *ad libitum*, at the breeding farm, to 100% of maintenance requirements in our experiment. The body weight loss that was recorded in the LU group during the last week of gestation, despite the increasing weight of the fetuses, is also reported elsewhere, for feeding levels similar to the ones in our experiment [15, 25, 26]. On the other hand, other researchers found that it takes a more severe maternal undernutrition (feeding levels less than 55 g/day) in order to achieve a significant reduction in the maternal body weight [14].

The feed intake of the does during gestation is also presented in table 2. Apart from the undernutrition periods, where the EU and LU groups consumed significantly less feed due to the dietary treatment (P<0.05), no other difference was observed between the experimental groups.

### Kindling performance and milk production of the does

**Fig 1:**
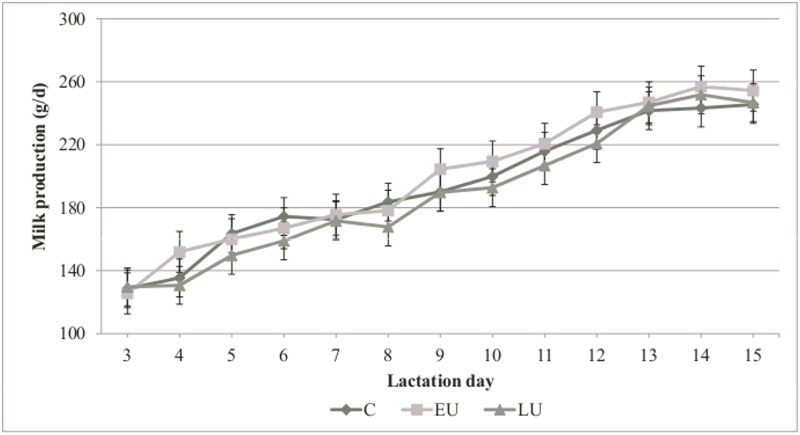
Effect of gestational undernutrition on milk production (g/d) of does at days 3–15 of lactation. C = does fed to 100% of maintenance energy requirements (n = 10); EU = does fed to 50% of maintenance energy requirements from day 7 to day19 of gestation (n = 8); LU = does fed to 50% of maintenance energy requirements from day 20 to day27 of gestation (n = 10). S.E. lines represent individual SEM.

The kindling performance of the does is presented in table 2. Gestation length, litter size and litter weight were not different among groups. In the EU group, two abortions were recorded, the first on gestational day 12 and the second on day 25 (premature birth). The C group had a significantly higher percentage of stillborn kits in comparison to the EU and LU groups (P<0.05) while the individual birth weight of the kits tended to be lower in the LU group (P<0.10). The average daily milk production of the experimental groups from day 3 to day 15 of lactation is presented in Fig. 1. The gestational maternal undernutrition had no effect on the milk production of the does (P>0.05).

The 50% feed restriction induced two abortions in our experiment (EU group). Feed restriction has been shown to induce abortion by other researchers as well [16, 26, 27], although not all studies have demonstrated a feed restriction-abortion link [25]. Therefore, the degree of feed restriction that a rabbit can sustain before abortion occurs has not been clearly determined.

The control group in our experiment produced a higher percentage of stillborn kits. This result has been previously reported for rabbits [28, 29] and more recently, it has been shown that *ad libitum* gestational feeding programmes of rabbit does have led to a higher toxaemia of females, increasing thus mortality of both females and new-born rabbit kits at peripartum [30]. Regarding the litter size at birth, it has been found that reduced gestational feeding levels do not affect the litter size at birth [16, 17, 31].

The LU group offsprings’ birth weight tended to be lower than the other two experimental groups. In earlier studies, with feeding levels similar to our own, fetal weight reductions were not produced [26, 27]. Nevertheless, this was not always the case since reduced fetal weights have been reported for higher [14] as well as lower feeding levels [25, 26, 27]. Probably, the critical factor is the doe’s physical and nutritional status before the gestation period. If it is good, a more severe undernutrition than 50% of energy requirements seems to be required in order to impair kit birth weight. In other farm and laboratory animals however, maternal undernutrition during gestation seems to have more detrimental effects on birth weight. In cattle [32], pigs [33], sheep [34], guinea-pigs [35] and rats [36] gestational feed restriction produced smaller offsprings as well as the partial limitation of the energy and/or protein intake.

### Growth performance of the offsprings

**Fig 2:**
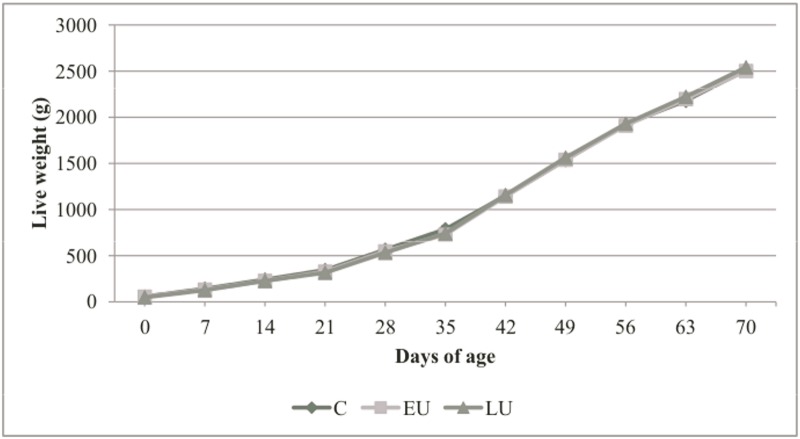
Effect of gestational maternal undernutrition on live weight (g) of rabbit offsprings from 0 to 70 days of age. C = rabbits born from does fed to 100% of maintenance energy requirements; EU = rabbits born from does fed to 50% of maintenance energy requirements from day 7 to day19 of gestation; LU = rabbits born from does fed to 50% of maintenance energy requirements from day 20 to day27 of gestation (n = 16). (Individual s.e. lines are present but not visible due to scale range).

The mean weekly body weights of the rabbits born from the control and undernourished groups from parturition to day 70 are presented in Fig. 2. No significant difference was observed between the groups at any age (P>0.05). The individual daily feed intake of the rabbits after weaning and their feed conversion ratio at 70 days of age was not different among groups (P>0,05). The average feed intake was 99 ± 4 g on wk 6, 145 ± 5 g on wk 7, 167 ± 6 g on wk 8 and 165 ± 5 g as well as 179 ± 5 g for wks 9 and 10, respectively. The average feed conversion ratio from weaning to slaughter was 3.01 ± 0.05.

The comparable growth of rabbit offsprings can be explained by the fact that the main factors that contribute to rabbit growth were more or less unaffected in our experiment. The rabbit’s growth is mainly dependent on two factors, birth weight and litter size, since it spends half of its life with the mother (gestation and lactation) [37]. In the present study, litter size was artificially equalized to 10 kits per doe shortly after parturition, in order to eliminate its effect and we recorded only a reduction tendency in birth weight. Such a small reduction in birth weight would have been eliminated by compensatory growth, a phenomenon well documented in rabbits, when *ad libitum* feeding is applied during the fattening period [38].

### Carcass and meat quality

Both carcass and meat quality of the offsprings were largely unaffected by the gestational maternal undernutrition (Tables 3 and 4, respectively). Carcass weights, carcass parts weights and internal organs weights were not different among groups (P>0.05). Moreover, no significant differences were recorded among groups on meat quality parameters of LL muscle.

**Table 3:** Effect of gestational maternal undernutrition on carcass quality parameters of rabbit offsprings (n = 16).

		Treatment^1^			
	C	EU	LU	pooled SE	p-value
Carcass weights (g)					
Live weight	2680	2664	2757	51	0.579
Hot Carcass	1732	1715	1755	28	0.801
Cold carcass	1690	1668	1705	27	0.840
Reference weight	1380	1357	1398	22	0.863
Dressing percentage (%)	63.2	62.6	61.9	0.8	0.650
Carcass parts weights (g)					
Head	143	142	143	3	0.854
Front	511	507	516	12	0.880
Middle	317	293	321	9	0.369
Rear	554	551	564	10	0.526
Hind leg	232	229	239	7	0.899
Fore leg	100	96	100	3	0.704
Internal organs weights (g)					
Liver	100	100	102	4	0.748
Perirenal fat	18	21	17	1	0.567
Kidneys	18	16	17	0.5	0.569
Lungs & heart	30	30	27	1	0.499
Spleen	1.7	1.5	1.7	0.1	0.680
Dissection weights (g)					
Fore leg					
Meat	64.1	61.5	60.2	2.4	0.447
Bone	18.8	17.6	15.9	0.7	0.689
Fat	4.3	7.1	6.7	1.3	0.565
Hind leg					
Meat	169	177	172	5	0.528
Bone	31.7	32.7	34.3	1.6	0.803
Fat	4.4	3.4	4.1	1.0	0.516

^1^ C = rabbits born from does fed to 100% of maintenance energy requirements;

EU = rabbits born from does fed to 50% of maintenance energy requirements from day 7 to day 19 of gestation;

LU = rabbits born from does fed to 50% of maintenance energy requirements from day 20 to day 27 of gestation.

**Table 4:** Effect of gestational maternal undernutrition on ***L*. *lumborum*** muscle meat quality characteristics of rabbit offsprings (n = 16).

		Treatment^1^			
n = 16	C	EU	LU	pooled SE	p-value
pH_24_	5.50	5.52	5.51	0.01	0.221
Colour^2^					
L*	59.6	59.0	59.5	0.5	0.734
a*	5.0	4.8	5.0	0.3	0.283
b*	13.6	13.7	13.6	0.2	0.892
PRW^3^ (%)	23.6	24.2	24.0	1.0	0.596
Cook Loss (%)	29.5	28.7	29.4	0.5	0.220
Shear values (N)	19.7	19.4	20.2	1.0	0.715

^1^ C = rabbits born from does fed to 100% of maintenance energy requirements;

EU = rabbits born from does fed to 50% of maintenance energy requirements from day 7 to day19 of gestation; LU = rabbits born from does fed to 50% of maintenance energy requirements from day 20 to day27 of gestation.

^2^ Colour:

L* = lightness,

a* = redness,

b* = yellowness

^3^ PRW = Percentage of released water.

Similar results have also been reported for lambs born from underfed ewes at 50% of the recommended allowance, and their birth weight was not impaired by the maternal undernutrition [39]. In other studies in pigs, where the birth weight of the offsprings was significantly reduced, the results were more impressive since both carcass composition and meat quality were clearly diminished [40, 41]. Therefore, there is a clear indication that the key parameter for detecting differences in carcass composition and meat quality after any type of maternal undernutrition is differences in birth weight.

## Conclusions

The gestational maternal undernutrition at the level of 50% of maintenance needs partially affected negatively the kindling performance of the does (abortions, tendency for lower birth weight), improved the liveability of the offsprings at birth and did not affect growth as well as carcass composition and meat quality of the offsprings. Therefore, it can be concluded that the undernutrition of the mother that is caused by the simultaneous lactation and gestation does not have detrimental effects on the productive and quality traits of the offsprings in rabbits. This is probably true, if the physical and nutritional status of the doe before the gestation period is good, otherwise more severe effects may be expected.
